# THE 4 S’S OF GERIATRICS EDUCATION: STIGMA, SPEED, STEALTH, AND SOURCES

**DOI:** 10.1093/geroni/igae098.1082

**Published:** 2024-12-31

**Authors:** Alissa Cooney, Edmund Duthie

**Affiliations:** Chair; Discussant

## Abstract

This symposium covers a conceptual framework developed by the Geriatrics Education Interest Group. The framework includes the 4 Ss of geriatrics education: Stigma, Speed, Stealth, and Sources. Geriatrics Educators have long needed the fortitude factor to face adversity in finding a place for geriatrics education in many curricula. Sharing the experience of geriatrics leaders who have demonstrated fortitude in their approach to geriatrics education fighting against stigma, getting creative with speed, seeking out stealthy opportunities, and leveraging sources, to improve geriatric education for all learners. The first S, Stigma, addresses the persistent ageist attitudes and behaviors found across healthcare by promoting geriatric education anchored in a “patient-centered care” approach addressing the specific, individual-based, concrete, patient care needs. The second S, Speed, explores micro learning in the context of geriatrics education to increase efficiency of learning and retention of knowledge. Multiple web-based platforms for geriatrics micro learning will be discussed including implementation of Geriatrics Fast Facts, a point-of-care micro learning resource. The third S, Stealth, shares how to reframe integration of geriatric principles specifically for non-geriatrics specialties. This segment will dive into case-based perspectives on stealthily integrating basic geriatric principles into learning environments not focused on geriatrics care. The fourth S, Sources, discusses how to leverage sources to enhance geriatric education. This section will explore the experience of geriatricians and inter professional geriatric specialists who have successfully leveraged their knowledge and resources and their institutions and local communities to benefit geriatric education. Geriatric Education Interest Group Sponsored Symposium

